# Proarrhythmia in a Patient With Heart Failure on Therapy With Amiodarone

**DOI:** 10.7759/cureus.15254

**Published:** 2021-05-26

**Authors:** Niya E Semerdzhieva, Ivo B Kozhuharov, Milko K Stoyanov, Christo G Tsekov

**Affiliations:** 1 Emergency Department, National Heart Hospital, Sofia, BGR; 2 Clinic of Cardiology, Department of Cardiac Stimulation, National Heart Hospital, Sofia, BGR; 3 Clinic of Cardiology, Department of Invasive Electrophysiology, National Heart Hospital, Sofia, BGR; 4 Surgical Clinic, National Transport Hospital 'Tzar Boris III', Sofia, BGR

**Keywords:** amiodarone, polymorphic ventricular tachycardia, heart failure, liver dysfunction, renal dysfunction

## Abstract

The deterioration of hepatorenal function due to worsening congestion is relatively common in acute heart failure and carries an independent adverse prognosis. In some patients, the risk of proarrhythmia is increased due to impaired drug metabolism. We described a patient with acute heart failure, polymorphic ventricular tachycardia (VT), and ventricular fibrillation episodes while receiving loading doses of amiodarone for atrial fibrillation. The occurrence of arrhythmia at the background therapy with a relatively safe antiarrhythmic drug in the settings of moderate cardiac, renal, and borderline liver functional impairment demonstrates that careful evaluation of liver and renal function is mandatory for the prevention of proarrhythmia.

## Introduction

Premature ventricular complexes, runs of nonsustained ventricular tachycardia (NSVT), and sustained ventricular tachycardia (VT) are common in patients with left ventricular (LV) dysfunction. They may be consequences of structural heart disease and may contribute to increased mortality risk. Optimized medical therapy for heart failure and amiodarone initiation are recommended in patients with LV dysfunction and VT [1]. Amiodarone is associated with a low incidence of proarrhythmia compared to other antiarrhythmic drugs [2]. However, amiodarone has no favorable effect on survival in patients with New York Heart Association (NYHA) class III heart failure and LV ejection fraction (EF) <35% [1].

The polymorphic VT torsades de pointes (TdP) is considered a proarrhythmia typically associated with amiodarone treatment. It has been observed in one-third of all proarrhythmic events in patients treated with this drug [2]. Physicians have a substantial lack of awareness regarding the risk of therapy with QT-prolonging drugs [3]. Abnormal liver or renal function are significant predictors of mortality in the population with heart failure [4], and we hypothesize that this is partly associated with delayed drug metabolism, excretion, and precipitating factors for fatal ventricular arrhythmia (VA).

## Case presentation

**Figure 1:**
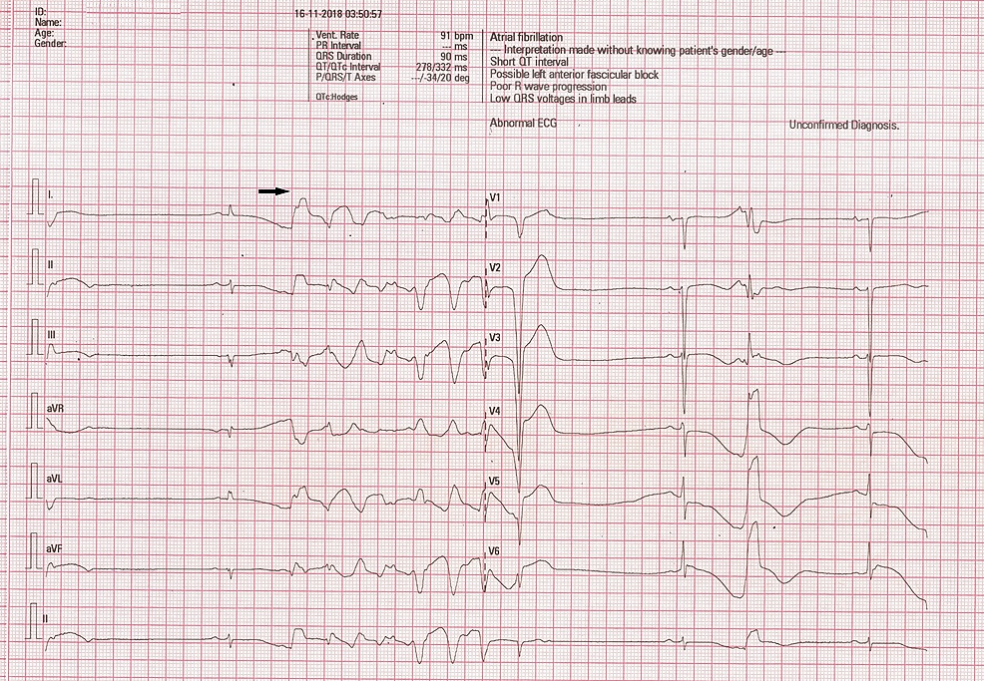
Electrocardiogram at the emergency ward.

А woman in her late 70s presented to the ED with chest discomfort and dyspnea with onset а few hours before presentation. She had a history of hypertension controlled by antihypertensive therapy, anemia, and stage IV renal disease. She was discharged from another hospital’s surgical clinic nearly one week earlier after being conservatively treated for ileus. She had undergone surgical resection of a benign tumor of the stomach more than five years ago. She was diagnosed with anemia in the surgical clinic. Following a transfusion of homologous blood, her hemoglobin was 99 g/L. She reported tightness in the chest and palpitations, and we detected paroxysmal atrial fibrillation, which necessitated amiodarone therapy. Her thyroid gland test results were within the reference range. The patient was discharged from the surgical clinic on therapy with amiodarone (she received a total of 4600 mg over eight days), bisoprolol (5 mg, twice), and ramipril 5 mg (twice). In our ED, she presented with orthopnea, pale skin, a blood pressure of 165/70 mmHg, and a pulse rate of 50 beats per minute (bpm). On cardiac auscultation, we noted muffled heart sounds and clear lungs. The serial electrocardiographic (ECG) records at admission showed sinus bradycardia with a ventricular rate of 47-54 bpm, prolongation in the corresponding QTc interval (462 to 508 msec), and runs of polymorphic NSVT (Figure 1).

An R-R interval of 1,800 msec preceded the polymorphic VT. In the next few days, the ECGs showed prolongation of the QTc interval up to 620 msec and T-wave inversion. Blood pressure-lowering therapy was started, and she was transferred to the intensive care unit (ICU). The patient received magnesium, and the rate and rhythm control medications were stopped. During the first few hours of her stay in the ICU, the patient had a ventricular fibrillation (VF) episode that necessitated an electric shock. Temporary transvenous pacing of her heart at 80 bpm was started to prevent future episodes of VT associated with prolonged QT in the setting of bradycardia. Her laboratory test results showed normal hemoglobin (128-131 g/L), leukocytosis (white blood cell count, 11.3 to 12.4 x109/L), renal dysfunction (glomerular filtration rate, 36.8-46.8 mL/min), total bilirubin of 18.6 mmol/L, marginally low potassium levels (3.5 mmol/L), and elevated cardiac-specific troponin I (50.5-56.5 ng/L). The initial echocardiographic examination showed moderately reduced LV EF (32%), global hypokinesia, severe mitral regurgitation (MR) and tricuspid regurgitation (TR), LV hypertrophy (13 mm), and marginally enlarged right ventricle (diameter of the base, focused four-chamber view: 42 mm). Cardiac catheterization and coronary angiography were performed due to suspicion of significant obstructive coronary disease and her recently diagnosed heart failure. The invasive study results confirmed some echocardiographic findings, including reduced EF (38%), global LV hypokinesia, severe MR and moderate TR, and LV end-diastolic pressure of 23 mmHg. Atherosclerotic plaques causing minor stenoses were found in all coronary arteries. A second echocardiographic study a few days later revealed mild MR and TR and 38% EF. The patient was informed on evidence-based therapy that can prevent the risk of sudden cardiac death: implantable cardioverter-defibrillator (ICD), which she declined. Given her persistent bradycardia, previous atrial fibrillation, and heart failure episodes, a permanent pacemaker was implanted and programmed to pace the heart at a rate of 70 bpm. The patient was released on therapy with bisoprolol 5+2.5 mg, furosemide 40 mg daily, spironolactone 25 mg once daily, magnesium aspartate tetrahydrate three times daily (500 mg), and pantoprazole 40 mg. She was assigned additional clinical examinations for anemia detected immediately before the current hospital admission.

The patient remained without symptomatic episodes of arrhythmia, presented with NYHA class II symptoms of heart failure, and was without anemia, according to laboratory findings at the end of the 28-month follow-up. Prophylaxis with anticoagulant medication has been initiated.

## Discussion

In this patient case, the evidence for proarrhythmia is the accumulation of several risk factors contributing to proarrhythmia: female sex, lost electrolyte balance (hypokalemia), bradycardia, and structural heart disease [5,6]. The patient had a recent transfusion of erythrocyte concentrate. Large blood transfusions are a rare cause of VA (VF) related mainly to subsequent low magnesium levels [6]. We have no available data on the acute plasma concentrations of magnesium and calcium in this patient. Magnesium and calcium levels are not tested routinely in the hospital’s laboratory. We could only speculate that the transfusion of erythrocytes and pre-existing electrolyte abnormalities (due to therapy with diuretics) contributed to the occurrence of polymorphic VA.

The occurrence of VA coincided with the effects of amiodarone on cardiac repolarization. These effects appear late-within at least 10 days of initiation [7]. The detected rhythm disturbance was observed when the drug’s loading dose was used concurrently with a high dose of the beta-blocker. A non-competitive block of beta receptors in the heart occurs within two days of the onset of amiodarone therapy [7]. When combined with rate-lowering or other antiarrhythmic drugs, amiodarone prolongs the QT interval and enhances the propensity for proarrhythmia and sinus arrest [8]. Finally, the assumption of proarrhythmia was supported by the fact that the observed rhythm disturbances disappeared after the beginning of the appropriate therapy [1].

Episodes of marked QT interval prolongation and VA also may frequently occur because of acute myocardial ischemia (80%) and less often in patients with advanced conduction system disease (heart block) and cardiomyopathy [1]. We excluded myocardial ischemia as a cause of arrhythmia in our patient using coronary angiography. A few types of proarrhythmia are associated with amiodarone intake and arise via a pathologic mechanism different from polymorphic VT TdP [2,9]. Our patient had structural heart disease. In experimental heart studies, wherein the late ion sodium current (INaL) is augmented to mimic acquired pathological conditions, amiodarone has a concentration-dependent biphasic effect of inducing and suppressing arrhythmia. Thus, patients with acquired pathological conditions (e.g., structural heart disease) associated with an increase in INaL may be at increased risk of proarrhythmia when the rectifying potassium ion current is inhibited by as little as 10%. At higher plasma concentrations, arrhythmic activity is suppressed, secondary to inhibition of both potassium ion and late sodium iron currents [9]. Although the arrhythmic activity of amiodarone is suppressed, secondary to inhibition of INaL, there is a strong positive association between the first episode of VF during the intake of amiodarone and the risk of death if the therapy continues [10]. The risk of proarrhythmia may vary in time in a patient with heart failure depending on the occurrence of precipitating factors for arrhythmia.

No renal side effects have been described concerning amiodarone therapy [11]. The deterioration of hepatorenal function during hospitalization is relatively common in acute heart failure and severe LV systolic dysfunction and carries an independent adverse prognosis [4]. Amiodarone can cause an asymptomatic rise of liver transaminases, symptomatic hepatotoxicity, and acute deterioration of the patient’s liver function in cases of pre-existing ischemia-induced liver damage [12,13]. One large study demonstrated that total bilirubin >17.1 μmol/L (>1 mg/dL) or creatinine >88.4 μmol/L (>1 mg/dL) or both concurrently were significant predictors of mortality in patients with heart failure [4,14]. Our case provides evidence that congestion in heart failure with moderately reduced EF results in renal and liver functional impairment severe enough to cause disturbed metabolism of amiodarone and proarrhythmia, despite the relatively safe cardiovascular profile of this antiarrhythmic.

The prophylactic insertion of a pacemaker to maintain a relatively high rate of 70-80 bpm, withdrawal of antiarrhythmic medication and magnesium, and potassium replenishment are appropriate acute treatments of TdP [1]. ICD therapy is recommended for patients with long QT syndrome who have survived a cardiac arrest [1]. In patients with nonischemic dilative cardiomyopathy, an ICD is recommended for those who experience hemodynamically unstable VT and cardiac arrest not due to reversible causes [1].

Another treatment approach, catheter ablation for VA in dilative cardiomyopathy is now increasingly used either primarily and/or combined with automatic anti-tachycardia devices and beta-blockers [15]. The different substrate of the arrhythmia - a reentry circuit in an area of myocardial scar rather than a focus of increased automaticity, and the related difficulties in defining the location of the reentry circuit and finding an effective ablation strategy in nonischemic dilative cardiomyopathy make the catheter ablation of polymorphic VT/VF a challenging procedure in this setting [15] that should be reserved for symptomatic patients with recurrent episodes.

Previous studies have reported varying results of the acute success of polymorphic VT ablation [16]. However, the catheter ablation of VF due to premature ventricular contractions in patients with long QT syndrome has been reported to have a high success rate with no recurrences of VF during a long-term follow-up period [17]. Recent data have confirmed that VF and VT may share circuitry. We do not know the exact mechanism of VT (increased automaticity or reentry circuit) in our patient; she declined electrophysiologic study. We discussed the possible irreversible abnormalities in repolarization related to myocardial structural and electrical remodeling in heart failure. The presence of structural heart disease favored the reentry mechanism of polymorphic VT in our patient’s case. The detection of both VT and VF further suggested a complex mechanism of arrhythmia in the described patient and indicated the risk of recurrent VA event with radiofrequency ablation treatment and the necessity of ICD implantation [18].

It is also important that transient conduction abnormalities could follow a radiofrequency procedure [19,20]. The slowing of conduction and heart rate may further predispose a patient to TdP VT, especially in the presence of other factors (subsequent beta-blockers for the treatment of nonischemic dilative cardiomyopathy; metabolic and electrolyte abnormalities related to therapy with diuretics). Therefore, the prophylactic maintaining of a relatively high rate of 70-80 bpm and the possibility of defibrillation (i.e., ICD implantation) of VT in case it recurs is also evidence-guided treatment for this case.

## Conclusions

Renal and liver congestion are common pathologic consequences of ventricular dysfunction in heart failure. The condition could be associated with impaired hepatic metabolism of amiodarone and risk of proarrhythmia. The evaluation and control of liver and renal function, as factors for proarrhythmia, are mandatory in the treatment of heart failure.
